# Serum soluble LYVE1 is a promising non-invasive biomarker of renal fibrosis: a population-based retrospective cross-sectional study

**DOI:** 10.1007/s12026-023-09448-3

**Published:** 2023-12-23

**Authors:** Jing Liu, Yuqing Liu, Wenqian Zhou, Yiguo Liu, Saiya Zhu, Ying Yu, Jieli Huang, Chen Yu

**Affiliations:** Department of Nephrology, Tongji Hospital, School of Medicine, Tongji University, No.389 Xincun Road, Putuo District, Shanghai, 200092 China

**Keywords:** Renal fibrosis, LYVE1, Lymphatic system, Immune, Lymphangiogenesis, Clinical trial

## Abstract

Diagnosis of renal fibrosis can only be verified by kidney biopsy, but biomarkers for non-invasive evaluation remain unsatisfactory. Patients with fibrosis often have abnormalities of the lymphatic vascular system and associated immune function. We describe here a lymphatic marker as a candidate biomarker for fibrosis. After assessing and grading the fibrosis scores, testing serum soluble lymphatic vessel endothelial hyaluronan receptor1 (sLYVE1) level, and collecting clinical information, the association between sLYVE1 and renal fibrosis was analyzed. Logistic regression analysis was used to screen variables. Diagnosis models with or without sLYVE1 were built, and nomograms were plotted. Calibration curve, C-index, and DCA were performed to assess the models. A total of 298 patients were enrolled in the study, of which 199 were included in the training cohort and 99 patients in the validation cohort. Serum sLYVE1 levels markedly elevated with increasing fibrosis grade (*p*<0.05). ROC analysis of sLYVE1 showed an AUC of 0.791 and 0.846 with optimal cut-off value of 405.25 ng/mL and 498.55 ng/mL for the prediction of moderate-to-severe renal fibrosis (MSF) and severe renal fibrosis (SF), respectively. The diagnostic nomogram model without sLYVE1 (model 1) included traditional clinical determinants (C-index: 0.658 for MSF; 0.603 for SF). A combination of model 1 and sLYVE1 (model 2) improved predictive performance (C-index: 0.847 for MSF; 0.856 for SF). Calibration curve and DCA demonstrated a better consistency accuracy and clinical benefit of model 2 than model 1. Serum sLYVE1 may be identified as a potential biomarker of renal fibrosis. Models incorporating sLYVE1 may be beneficial for a more accurate non-invasive diagnosis of renal fibrosis.

**Supplementary Information:**

The online version contains supplementary material available at 10.1007/s12026-023-09448-3.

## Introduction

Renal fibrosis, characterized by arteriolosclerosis, glomerulosclerosis, interstitial fibrosis, and tubular atrophy, has been regarded as the ultimate manifestation of chronic kidney disease (CKD) to end-stage kidney disease (ESKD) [1]. A timely diagnosis of fibrosis, which currently relies heavily on renal biopsy, is crucial. However, the invasive nature of kidney biopsies hinders the possibility of performing clinical examinations, especially secondary or multiple examinations. Currently, estimated glomerular filtration rate (eGFR) and albuminuria are generally recognized as standard biomarkers for predicting CKD risk [2]. However, eGFR and proteinuria often represent glomerular damage, and tubulointerstitial fibrosis is more relevant to the progression of renal fibrosis than glomerulosclerosis [3]. Hence, there is an urgent requirement for new surrogate biomarkers that can capture the extent and evolution of fibrosis more conveniently and accurately.

The lymphatic and blood vascular systems constitute the circulatory system, working together to maintain body homeostasis. The renal lymphatic system is located mainly in the cortex and originates from capillary lymphatic vessels (LVs) and finally converging to the renal drainage lymph nodes [4]. Besides interstitial fluid and lipid, immune response participants, such as antigens and immune cells, are also transported through LVs. Alterations in the composition of these lymphatic fluids in the tissues, like tissue injury, inflammation, and interstitial fluid overload, can lead to abnormalities in the lymphatic system. Although not as well investigated as the glomerulus and tubules, several studies have emerged in recent years on the association of kidney disease with the lymphatic system. Multiple pathological conditions including inflammation (acute/chronic kidney injury, transplantation) and excess interstitial fluid (hypertension) tend to provoke functional and quantitative expansion of LVs [5]. For renal fibrosis, fibrosis severity-related lymphangiogenesis has been demonstrated in rodent models and in patients with CKD [6–8]. In these research, lymphangiogenesis or lymphatic invasion was detected by lymphangiogenic marker vascular endothelial growth factor C or LVs marker lymphatic vessel endothelial hyaluronan receptor1 (LYVE1) [9]. LYVE1 is selectively expressed on the endothelium of lymphatic capillaries and is therefore to be regarded as one of the potent markers of LVs [10]. While research into increased local renal LYVE1 expression in renal fibrosis has flourished [9], the literature on the relationship between fibrosis and circulating LYVE1 remains unexplored. Moreover, since lymphangiogenesis is time- and severity-dependent [6–8], it is worthwhile to investigate whether circulating LYVE1 can represent the degree of lymphangiogenesis and thus reflect renal lesions. In fact, previous studies have reported the connection of serum soluble LYVE1 (sLYVE1) level with lymphoproliferative diseases such as cancer and rheumatoid arthritis. In clinically unremitting rheumatoid arthritis patients, serum LYVE1 levels were positively correlated with CRP [11]. In contrast, Banerji, S et al. found that serum LYVE1 was significantly lower in patients with metastatic lung cancer than in patients without metastatic lung cancer [12].

Given the strong relationship between the lymphatic system and renal fibrosis, a correlation between serum sLYVE1 levels and the severity of renal fibrosis was hypothesized. Here, we assessed whether serum sLYVE1 could serve as a latent biomarker of renal fibrosis and further explored and validated models by including (or not) sLYVE1 for patients.

## Methods

### Study design and populations

This retrospective observational study was done in Shanghai Tongji Hospital and was approved by the ethics committee of Tongji Hospital (Shanghai, China; approval NO.: K-W-2021-003). Patients included in this study have signed informed consent to use their samples for subsequent clinical studies. We enrolled patients who underwent renal biopsy at Shanghai Tongji Hospital from January 2016 to April 2023 and screened patients according to the following exclusion criteria: (1) <18 or >80 years old; (2) patients with missing blood samples; (3) acute kidney injury; (4) patients with malignant cancer, severe liver failure, heart failure, respiratory failure, pulmonary fibrosis, hepatic fibrosis, or scleroderma (Supplement figure 1).

### Clinical characteristics

Demographic characteristics (i.e., gender and age) and traditional laboratory assessments of patients were collected from the electronic medical records. The results of traditional clinical characteristics, including blood urea nitrogen (BUN), serum creatinine (sCr), urine acid levels (UA), serum cystatin c (CyC), blood superoxide dismutase (SOD), serum albumin (ALB), 24-h urinary protein level, and estimated glomerular filtration rate (eGFR), serum kappa, and lambda light chain, were carried out in the clinical laboratory of Tongji Hospital within 2 days before biopsy. Due to the low impact of CyC on diet and muscle, eGFR was calculated with cystatin C-based equations (CKD-EPI-CyC) [13, 14]. Nephrotic-range proteinuria (NS-proteinuria) was typically defined as daily urine protein >3g [15], and hypoalbuminemia was defined as concentration< 30g/L [16].

Renal biopsies were performed by ultrasound-guided percutaneous puncture and subsequently fixed in 10% formalin as previously described [17]. Light microscopy was used for histologic diagnosis, and fibrosis was scored at this time point in a double-blind manner by two renal pathologists based on the Banff classification [18] and the total renal chronicity score proposed by Sethi S et al. [1], on a scale ranging from non to severe fibrosis. In cases where the two ratings differed, a third pathologist gave the rating independently. In brief, arteriosclerosis or arteriolosclerosis (scored from 0 to 1), global and segmental glomerulosclerosis (scored from 0 to 3), interstitial fibrosis (scored from 0 to 3), and tubular atrophy (scored from 0 to 3) were evaluated, and the sores were added together to stage the overall grade of renal fibrosis.

### Specimen collection and sLYVE1 measurement

The same procedure was followed for all sample collections. Blood samples were collected intravenously early in the morning of the second day of the patient’s admission, and renal puncture was performed on the same day. Blood samples were centrifuged, transferred to freezing tubes, and stored at −80°. After screening based on exclusion criteria, patient blood samples were retrospectively selected for the next step of testing. The samples were thawed, and sLYVE1 concentrations were examined with a commercial ELISA kit (Cusabio, Wuhan, China) according to instructions.

### Statistical analysis

For descriptive statistics, frequencies (proportions) were used to report categorical variables, while continuous variables were expressed as medians (IQRs) or means ± SDs. Missing data were addressed by multiple imputation. Differences between medians or means were determined by unpaired *t*-test and between frequencies (proportions) with the *x*² test.

Patients were assigned to the training and the validation cohort in a 2:1 ratio using computer-generated random numbers. In the training cohort, univariate logistic analysis was used to identify clinically relevant variables associated with the prediction of renal fibrosis and to calculate odds ratios (ORs) as well as 95% CIs. Statistically significant variables were enrolled to develop the final models and plot corresponding nomograms. To evaluate the discrimination and calibration of the nomogram, receiver operating characteristic (ROC) curves with area under curve (AUC) and calibration curves were used respectively. Also, decision curve analyses (DCA) were performed to assess the clinical benefit of models.

All statistical analyses were carried out with Rstudio (version 2022.07.0+548), and *p*<0.05 was considered to indicate a statistically significant difference.

## Results

### Study populations

From January 2016 to April 2023, 298 patients were eligible for inclusion in the research, 179 (60.07%) of these patients were male. For the fibrosis grade, 101 (33.89%) had no renal fibrosis, 100 (33.56%) had mild renal fibrosis, 55 (18.46%) had moderate renal fibrosis, and the other 42 (14.09%) patients had severe fibrosis (Table 2). IgA nephropathy, membranous nephropathy, and diabetic nephropathy were the most common pathological types in the cohort, with 72 (23.83%), 67 (22.48%), and 34 (11.41%) individuals, respectively. Other baseline characteristics are shown in Table 1 and Table 2.

**Table 1:** Baseline characteristics and biochemical measurements of patients enrolled

Characteristics		
Baseline features	Male	179 (60.07)
	Age (year)	50.00 (35.00, 64.00)
Laboratory examinations	sLYVE1 (ng/mL)	405.71 (294.53, 540.06)
	Cystatin C (mg/L)	1.55 (1.13, 2.49)
	SOD (U/mL)	109.22 (81.80, 143.78)
	Serum albumin (g/L)	31.10 (22.70, 36.90)
	Urea (mmol/L)	6.78 (5.09, 10.71)
	UA (μmol/L)	423.00 (356.70, 519.00)
	sCr (μmol/L)	97.00 (71.00, 158.50)
	Estimated GFR (mL/(min*1.73m^2))	61.50 (39.88, 85.72)
	24 h urine protein (g/24h)	2.76 (1.12, 7.76)
Disease evaluation	No fibrosis	101 (33.89)
	Mild fibrosis	100 (33.56)
	Moderate fibrosis	55 (18.46)
	Severe fibrosis	42 (14.09)
Pathology	IgA nephropathy	70 (23.49)
	Membranous nephropathy	67 (22.48)
	Diabetic nephropathy	34 (11.41)
	Minimal change disease	30 (10.07)
	Renal arteriosclerosis	21 (7.05)
	Focal segmental glomerulosclerosis	15 (5.03)
	Lupus nephritis	13 (4.36)
	Proliferative glomerulonephritis	10 (3.36)
	Other	38 (12.75)

*sCr*, serum creatinine; *GFR*, glomerular filtration rate; *sLYVE1*, soluble LYVE1; *SOD*, superoxide dismutase; *UA*, uric acid

**Table 2:** Baseline clinical characteristics and biochemical measurements in patients classified by fibrosis grade

Characteristics		Non	Mild	Moderate	Severe
Baseline features	Male	49 (48.51)	58 (58.00)	33 (60.00)	38 (90.48)
	Age (year)	46.00 (33.00, 65.00)	52.00 (33.00, 64.00)	49.00 (37.00, 58.00)	48.00 (36.00, 64.00)
Laboratory examinations	sLYVE1 (ng/mL)	340.42 (255.87, 519.92)	331.82 (238.79, 449.33)	467.83 (365.68, 547.89)	609.22 (510.45, 752.74)
	Cystatin C (mg/L)	1.31 (1.03, 2.32)	1.23 (1.08, 1.70)	1.90 (1.51, 2.43)	2.91 (1.65, 3.60)
	SOD (U/mL)	111.10 (79.90, 147.30)	106.30 (84.80, 151.10)	114.40 (97.90, 140.30)	106.60 (81.10, 129.40)
	Serum albumin (g/L)	29.40 (21.40, 36.00)	30.20 (20.90, 38.00)	35.30 (29.05, 39.80)	30.40 (24.10, 35.20)
	Urea (mmol/L)	5.51 (4.59, 8.75)	5.82 (4.61, 8.06)	8.74 (6.63, 11.58)	14.86 (10.73, 24.36)
	UA (μmol/L)	414.03 (324.48, 511.33)	403.52 (350.53, 498.06)	488.02 (412.00, 553.23)	477.00 (386.50, 528.00)
	sCr (μmol/L)	78.10 (66.00, 107.20)	78.50 (65.50, 106.80)	140.00 (106.00, 169.50)	282.00 (171.00, 529.50)
	Estimated GFR-EPI (mL/(min*1.73m^2))	72.60 (42.01, 94.78)	74.69 (54.00, 90.13)	52.61 (40.69, 63.32)	36.43 (31.37, 59.15)
	24 h urine protein (g/24 h)	2.49 (0.72, 8.24)	2.37 (0.94, 6.78)	2.57 (1.37, 6.60)	4.85 (1.90, 9.90)

*sCr*, serum creatinine; *GFR*, glomerular filtration rate; *sLYVE1*, soluble LYVE1; *SOD*, superoxide dismutase; *UA*, uric acid

### sLYVE1 was associated with renal fibrosis

**Fig. 1:**
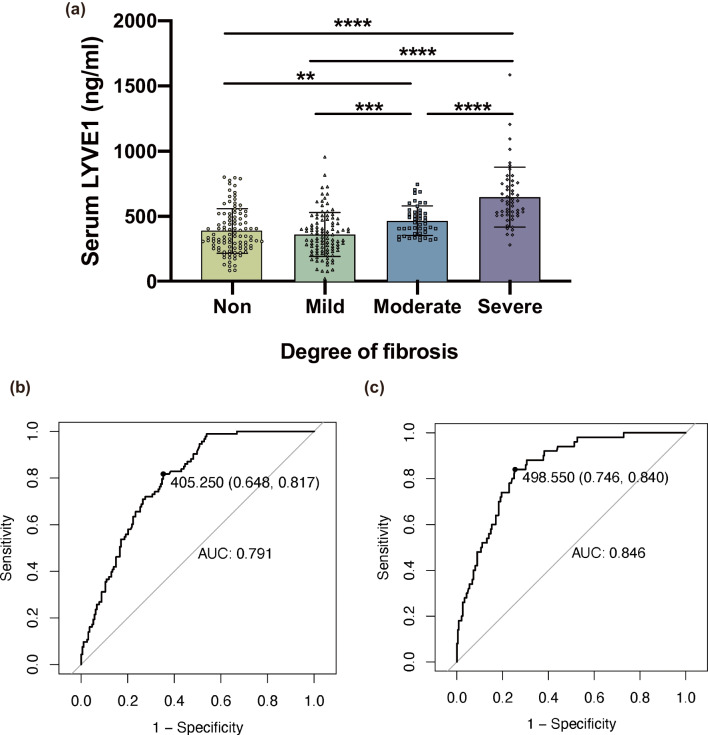
**a** Serum sLYVE-1 levels in relation to grade of renal fibrosis. Results are presented as mean ± SD. One-way ANOVA was performed to compare differences between groups. **b** ROC curve of sLYVE1 to predict moderate-to-severe fibrosis. **c** ROC curve of sLYVE1 to predict severe fibrosis

A total of 2 patients had serum sLYVE1 concentrations outside the range of detection of the standard curve, and this missing value was addressed using multiple imputation. To assess the relationship between sLYVE1 and renal fibrosis, subjects were divided into four groups according to Banff classification. Notably, sLYVE1 levels rose with increasing grade of renal fibrosis. In detail, compared with the non-fibrosis (340.42 (255.87, 519.92)) or mild fibrosis (331.82 (238.79, 449.33)) groups, sLYVE1 concentrations were significantly increase in the moderate (467.83 (365.68, 547.89)) or severe fibrosis (609.22 (510.45, 752.74)) groups, and sLYVE1 expression was also significantly different between the moderate and severe fibrosis groups. However, there was no significant difference in non-fibrosis group and mild fibrosis group, which is consistent with the fact that lymphangiogenesis tends to occur in the middle to late stages of renal fibrosis (Fig. 1a). Therefore, next we focused on the diagnostic ability of sLYVE1 for moderate to severe fibrosis (MSF) and severe fibrosis (SF), with mild fibrosis being excluded from the exploration.

The receiver operating characteristic curve (ROC) was plotted to search for a diagnostic cut-off of serum sLYVE1 for MSF and SF. Area under the curve (AUC) was calculated as 0.791 (95% CI 0.648–0.817) and 0.846 (95% CI 0.746, 0.840) with cut-off values of 405.25 ng/mL and 498.55 ng/mL, respectively, in MSF and SF group (Fig. 1b, c).

**Fig. 2:**
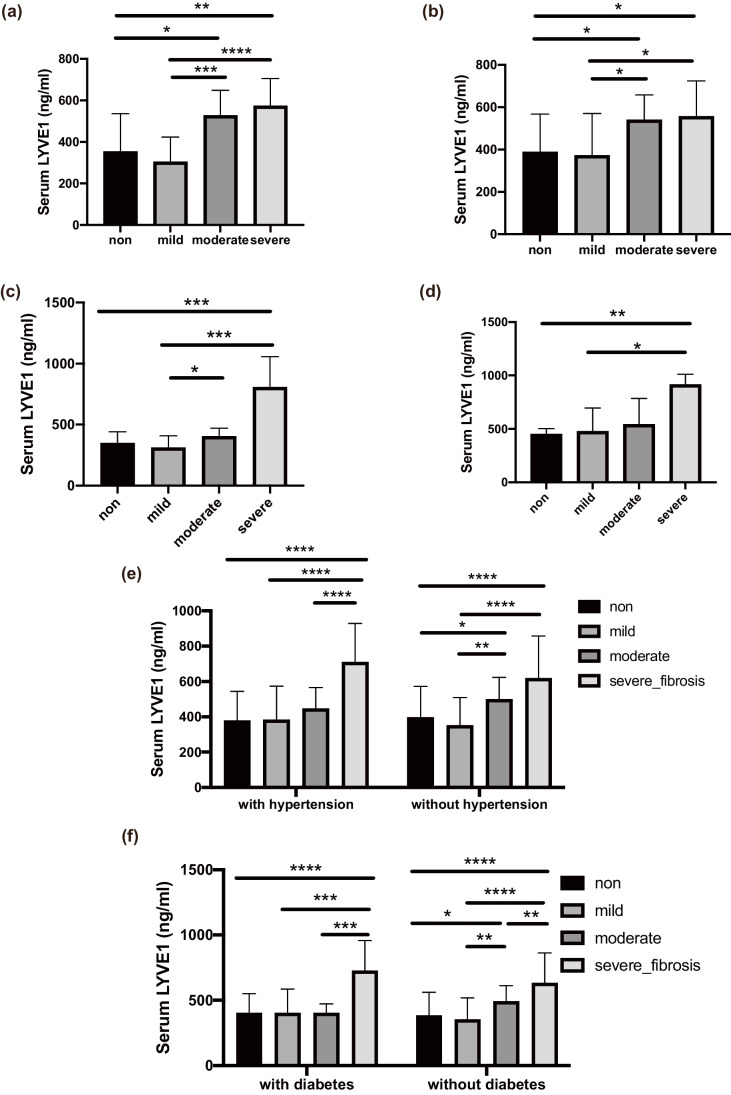
**a**–**d** Subgroup analysis according to different pathology types: **a** IgA nephropathy; **b** membranous nephropathy; **c** diabetic nephropathy; **d** lupus nephritis. **e**, **f** Subgroup analysis according to complications: **e** hypertension; **f** diabetes

Further subgroup analyses based on renal pathology and complications were also performed. In the IgA nephropathy, membranous nephropathy, diabetic nephropathy, and lupus nephritis subgroups, sLYVE1 levels were higher in patients with moderate and severe fibrosis than in patients in the non-mild and mild groups, irrespective of pathology type (Fig. 2a–d). Similarly, in subgroup analyses based on the two most common comorbidities (hypertension and diabetes mellitus), sLYVE1 also both increased with increasing fibrosis degree (Fig. 2e, f).

### Elevated serum sLYVE1 was not linked to decreased renal function

**Fig. 3:**
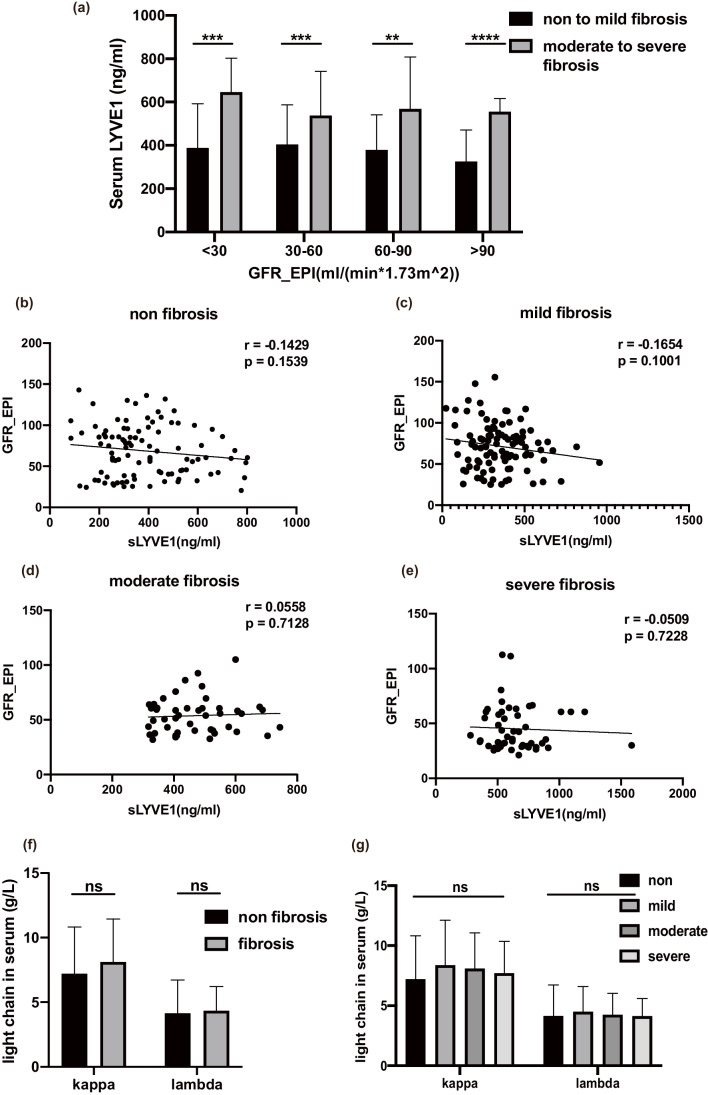
**a** Serum sLYVE1 levels were higher in patients with moderate-to-severe fibrosis than in patients with non-mild fibrosis, independent of GFR classification. **b**–**d** In all four fibrosis classes, sLYVE1 was not associated with GFR. **e**, **f** Serum kappa and lambda light chains did not significantly change with renal fibrosis

To clarify that the elevation of sLYVE1 was indeed due to fibrosis and not to circulating accumulation of serum small molecules due to impaired renal function, we performed a stratified analysis based on GFR. Serum sLYVE1 levels were significantly higher in patients with moderate-to-severe fibrosis than in patients with non-to-mild fibrosis in all subgroups with different GFR levels (Fig. 3a). Furthermore, no correlation between sLYVE1 and GFR was found after classifying patients according to fibrosis grade (Fig. 3b–e). Serum light chains have a similar molecular weight to LYVE1, and their levels correlate with abnormal plasma cell function rather than fibrosis, making them an ideal control for this study. Stratified analyses based on the presence or absence of fibrosis (Fig. 3f) or grade of fibrosis (Fig. 3g) showed no significant changes in the kappa light chains and λ light chains of the patients. These results demonstrate the increase in sLYVE1 levels is due to fibrosis rather than impaired renal filtration function.

### Feature selection and nomogram models

Since ROC curve analysis showed that serum sLYVE1 alone was a good indicator in determining MSF and SF, we moved on to investigate whether it could assist the diagnostic ability of traditional clinical tests. Therefore, a total of 298 patients were randomly divided into a training cohort and a validation cohort by a ratio of 2:1. As shown in Table 3, there were no statistical differences in baseline clinical indicators between the 2 cohorts.

**Table 3:** Baseline characteristics and biochemical measurements of two cohorts

Characteristics		Training set (*n*=199)	Validation set (*n* = 99)	*P* value
Baseline features	Male	117 (58.79)	62 (62.63)	0.525
	Age (year)	51.00 (34.00, 64.00)	48.00 (36.00, 63.00)	0.863
Laboratory examinations	sLYVE1 (ng/mL)	405.72 (283.38, 534.91)	405.58 (317.38, 555.86)	0.250
	Cystatin C (mg/L)	1.52 (1.10, 2.45)	1.60 (1.14, 2.54)	0.087
	SOD (U/mL)	116.20 (84.70, 145.40)	98.65 (78.65, 136.42)	0.207
	Serum albumin (g/L)	31.70 (22.40, 37.00)	29.90 (22.65, 36.68)	0.569
	Urea (mmol/L)	6.50 (5.01, 10.67)	7.21 (5.16, 11.38)	0.572
	UA (μmol/L)	416.04 (356.32, 515.00)	463.48 356.71, 527.29)	0.157
	sCr (μmol/L)	94.00 (71.00, 153.00)	106.50 (71.00, 165.80)	0.988
	Estimated GFR-EPI (mL/(min*1.73m^2))	62.55 (40.93, 86.66)	60.64 (39.68, 83.98)	0.376
	24 h urine protein (g/24 h)	3.01 (1.11, 7.23)	2.48 (1.10, 8.81)	0.773
Disease evaluation	No fibrosis	68 (34.17)	33 (33.33)	0.795
	Mild fibrosis	67 (33.67)	34 (34.34)	0.908
	Moderate fibrosis	35 (17.59)	20 (20.20)	0.584
	Severe fibrosis	29 (14.57)	12 (12.12)	0.563
Pathology	IgA nephropathy	50 (25.13)	20 (20.20)	0.594
	Membranous nephropathy	44 (22.11)	23 (23.23)	0.827
	Diabetic nephropathy	22 (11.06)	12 (12.12)	0.785
	Minimal change disease	20 (10.05)	10 (10.10)	0.989
	Renal arteriosclerosis	13 (6.53)	8 (8.08)	0.623
	Focal segmental glomerulosclerosis	9 (4.52)	6 (6.06)	0.567
	Lupus nephritis	9 (4.52)	4 (4.04)	0.848
	Proliferative glomerulonephritis	6 (3.02)	4 (4.04)	0.643
	Other	26 (13.07)	12 (12.12)	0.818

*sCr*, serum creatinine; *GFR*, glomerular filtration rate; *sLYVE1*, soluble LYVE1; *SOD*, superoxide dismutase; *UA*, uric acid

### Predictive models incorporating sLYVE1 had better performance for MSF

The results of the stepwise regression suggested that the model consisting of sLYVE1, gender, GFR, and hypoalbuminemia had the lowest AIC value and VIF value below 3, indicating no collinearity between variables in the model. Binary logistic regression confirmed that high sLYVE1, males, and hypoalbuminemia were independent predictors of MSF and were associated with an increased risk of MSF (Table 4). Although the indicator of nephrotic syndrome-level-proteinuria (NS-proteinuria) had a *p*-value of >0.05 in the univariate logistic regression analysis, it was also included in the model due to its importance in clinical diagnosis.

**Table 4:** Results of logistic regression analysis of clinical indicators for moderate-to-severe fibrosis

Variables	*B*	Wald	Exp (*B*)	Exp (*B*) 95% CI	*P* value
Age	0.007	0.598	1.009	0.990, 1.027	0.356
Gender	0.871	7.036	2.314	1.228, 4.360	0.009
sLYVE1	0.05	24.213	1.052	1.031, 1.073	<0.001
Cystatin C	0.660	11.386	1.983	1.352, 2.908	<0.001
SOD	−0.005	2.095	0.994	0.984, 1.004	0.226
Hypoalbuminemia	0.489	3.511	1.99	1.051, 3.757	0.035
Urea	0.115	22.193	1.123	1.070, 1.178	<0.001
UA	0.005	13.272	1.005	1.003, 1.008	<0.001
sCr	0.009	19.252	1.009	1.005, 1.013	<0.001
GFR	−0.042	12.389	0.966	0.955, 0.978	<0.001
NS level proteinuria	0.318	1.581	1.374	0.751, 2.513	0.302

*sCr*, serum creatinine; *GFR*, glomerular filtration rate; *sLYVE1*, soluble LYVE1; *SOD*, superoxide dismutase; *UA*, uric acid

**Fig. 4:**
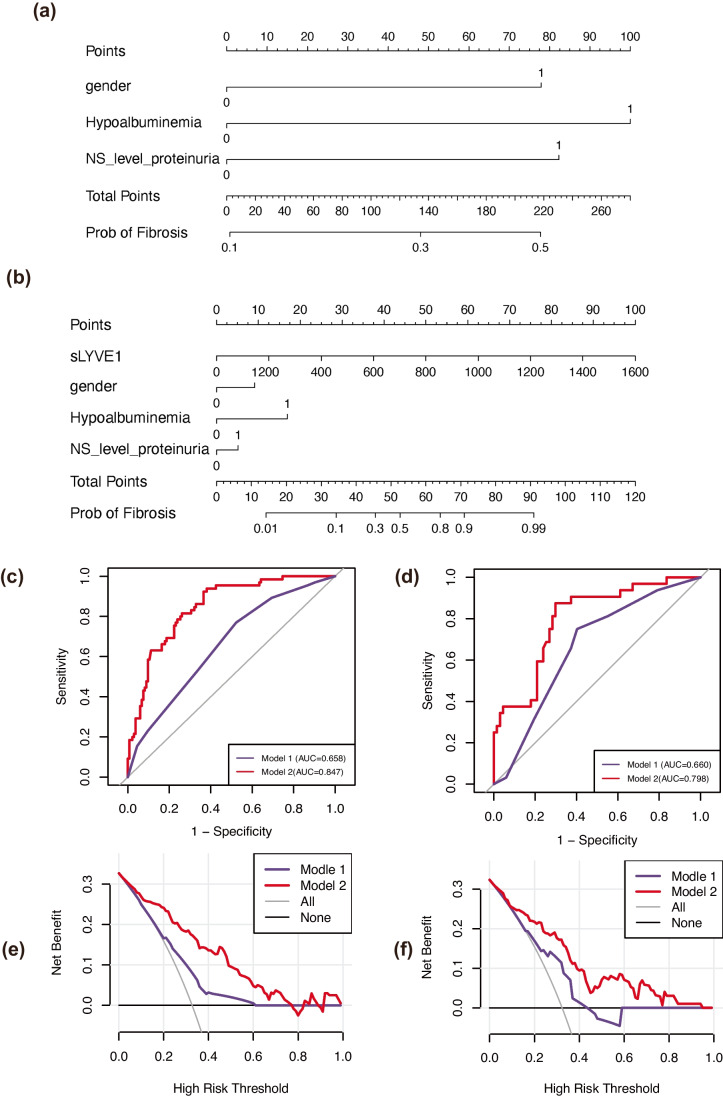
Models of predicting moderate-to-severe fibrosis. **a**, **b** Nomogram of models 1 (without sLYVE1) and 2 (with sLYVE1); **c** ROC curves of two models in the training cohort. **d** ROC curves of two models in the validation cohort. **e** DCA curves of two models in the training cohort. **f** DCA curves of two models in the validation cohort

To assess whether sLYVE1 enhances the diagnostic power of traditional clinical indicators, two models were developed. Model 1 consisted of traditional clinical indicators only, while model 1 incorporated sLYVE1 constituted model 2. Nomograms of models 1 and 2 are plotted in Fig. 4a and b.

We then validated the nomograms with ROC curves and calibration curves and used DCA curves to assess their clinical value. The calibration curves demonstrated good consistency in both the training cohort (Supplementary Fig. 2a, 2b) and the validation cohort (Supplementary Fig 2c, 2d). The C-index for model 2 was higher than model 1 in both the training (0.847 vs. 0.658) and validation sets (0.798 vs. 0.660), indicating better accuracy for model 2 (Fig. 4c, d). Also, DCA curves of model 2 exhibited larger net clinical benefits than model 1 in two cohorts (Fig. 4e, f).

### Predictive models incorporating sLYVE1 had better performance for SF

**Fig. 5:**
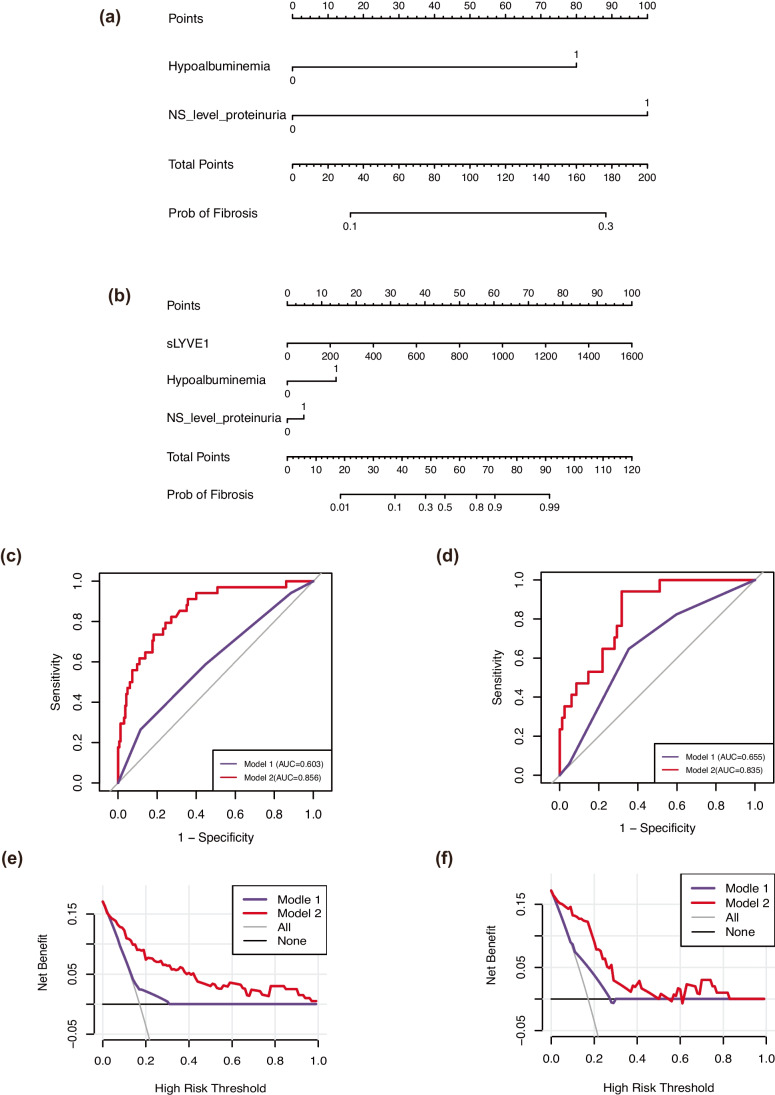
Models of predicting severe fibrosis. **a**, **b** Nomogram of models 1 (without sLYVE1) and 2 (with sLYVE1); **c** ROC curves of two models in the training cohort. **d** ROC curves of two models in the validation cohort. **e** DCA curves of two models in the training cohort. **f** DCA curves of two models in the validation cohort

To explore the diagnostic value of sLYVE1 as an aid in SF, we performed a similar assessment to that in MSF. Since the model with sLYVE1, hypoalbuminemia had minimal AIC value and low VIF (Table 5), they were incorporated into the models (Fig. 5a, b).

**Table 5:** Results of logistic regression analysis of clinical indicators for severe fibrosis

Variables	*B*	Wald	Exp (*B*)	Exp (*B*) 95% CI	*P* value
Age	0.015	1.463	1.015	0.991, 1.038	0.223
Gender	0.624	2.310	1.973	0.887, 4.388	0.096
sLYVE1	0.062	25.098	1.066	1.040, 1.092	<0.001
Cystatin C	0.788	14.139	2.277	1.510, 3.435	<0.001
SOD	−0.006	0.804	0.992	0.979, 1.006	0.250
Hypoalbuminemia	0.096	0.091	1.252	0.582, 2.694	0.565
Urea	0.118	27.707	1.124	1.076, 1.175	<0.001
UA	0.004	4.668	1.004	1.002, 1.007	0.024
sCr	0.007	25.049	1.007	1.005, 1.010	<0.001
GFR	−0.049	8.743	0.960	0.945, 0.977	<0.001
NS level proteinuria	0.852	7.086	1.849	0.871, 3.924	0.110

*sCr*, serum creatinine; *GFR*, glomerular filtration rate; *sLYVE1*, soluble LYVE1; *SOD*, superoxide dismutase; *UA*, uric acid

Calibration curves and DCA curves were displayed in Fig. 5c–f and Supplementary Fig 3, indicating that the model had a better consistency accuracy and greater net clinical benefits with the inclusion of sLYVE1. Furthermore, the results of ROC analysis revealed a better discriminate power of model 2 than model 1 in two sets (training set: 0.856 vs. 0.603; validation set: 0.835 vs. 0.655).

## Discussion

Abnormalities in the number and function of LVs are often present in renal fibrosis. Although LYVE1 has been widely used as an LV marker and its increase signifies LVs proliferation, the role of circulating sLYVE1 in renal fibrosis has not been investigated. The current study provides new insights into the predictive function of sLYVE1 on fibrosis by showing (1) serum sLYVE1 levels were markedly higher in moderate and severe fibrosis groups than non and mild fibrosis groups. (2) sLYVE1 is an independent predictor of MSF and SF. (3) sLYVE1 can complement traditional clinical indicators for the diagnosis of renal fibrosis (Tables 6 and 7).

**Table 6:** Prediction models with or without sLYVE1 for moderate-to-severe renal fibrosis patients

Factors		Model 1			Model 2		
		*β*	OR (95%CI)	*P* value	*β*	OR (95%CI)	*P* value
Traditional clinical features	Gender	0.794	2.079 (1.178, 3.667)	0.012	0.977	2.656 (1.315, 5.367)	0.006
	Hypo-albuminemia	1.019	2.723 (1.329, 5.579)	0.006	1.504	4.501 (1.877, 10.789)	<0.001
	NS level proteinuria	0.839	2.389 (1.178, 4.846)	0.016	0.690	1.993 (0.891, 4.457)	0.093
New feature	sLYVE1	NA	NA		0.066	1.068 (1.046, 1.091)	<0.001
C-index	Training cohort	0.658			0.847		
	Validation cohort	0.660			0.798		

*NS*, nephrotic syndrome; *sLYVE1*, soluble LYVE1

**Table 7:** Prediction models with or without sLYVE1 for severe renal fibrosis patients

Factors		Model 1			Model 2		
		*β*	OR (95%CI)	*P* value	*β*	OR (95%CI)	*P* value
Traditional clinical features	Hypo-albuminemia	0.753	2.124 (1.042, 4.791)	0.047	1.185	3.270 (1.259, 8.495)	0.015
	NS level proteinuria	1.305	3.687 (1.620, 8.392)	0.002	0.952	2.592 (1.014, 6.625)	0.047
New feature	sLYVE1	NA	NA		0.072	1.075 (1.049, 1.101)	<0.001
C-index	Training cohort	0.603			0.856		
	Validation cohort	0.655			0.835		

*NS*, nephrotic syndrome; *sLYVE1*, soluble LYVE1

Sharing a 44% sequence similarity with the vascular hyaluronic acid (HA) receptor CD44, LYVE1 is a 340-residue transmembrane receptor containing an HA-binding link module in the extracellular domain. On the basis of this specific structure, LYVE1 is involved in HA metabolism and in the transport of immune cells expressing HA [19], thus mediating the immune regulation of organs in physiological and pathological states. Although LYVE1 has long been recognized as an LV marker, it is only since the work of Nunomiya K [12] that studies of circulating soluble LYVE1 in pathological situations have gained momentum, albeit minimally. Their retrospective study found that sLYVE1 was negatively correlated with tumor size as well as metastasis. Then, Yoo J et al. reported a positive correlation between sLYVE1 and C-reactive protein in the non-clinical remission group of patients with rheumatoid arthritis [11]. The disease features of renal fibrosis share many similarities with rheumatoid arthritis and malignancy, including a chronic inflammatory response [20] and enlargement of the lymphatic system [7, 21] that occurs late in the disease progression. Our data clearly show a dramatic increase in serum sLYVE1 expression in MSF and SF patients, but no variation in mild renal fibrosis. This dynamic change correlates closely with the time point of LVs proliferation in renal fibrosis. Yazdani S [22] set up a time course study, monitoring sites every 6 weeks from 6 weeks to 30 weeks to investigate the timing of new LVs formation in a rat model of adriamycin-induced renal fibrosis and observed a significant increase in the number of LVs starting at 12 weeks and continuing until the end of the study. These observations are compatible with the finding that significant lymphangiogenesis only occurs in patients with grade 2–3 fibrotic lesions [8]. Taken together, the results of these previous studies and our data suggest that increased serum sLYVE1 may reflect changes in LVs in the kidney and the severity of disease in the mid-to-late stage of fibrosis.

Although a kidney biopsy can show damage at any level, including the tubules, glomeruli, and vascular system, it is an invasive procedure, which is a major concern for patients who abandon it. Therefore, the identification of non-invasive biomarkers capable of estimating dynamic changes in renal fibrosis will be of fundamental importance. Indeed, several serum biomarkers have been used to assess renal fibrosis, although still scattered. Of these, markers reflecting activated monocytes/macrophages [23], renal tubular injury [24], collagen formation [25, 26], and the matrix metalloprotease family [27] are the most studied, although most are not confirmed histologically [28]. While not mentioned in this study, enhanced expression of renal LYVE1 in fibrosis has been demonstrated in numerous studies using immunoblotting and immunohistochemical staining [9], providing a histological basis for our study. Increased sLYVE1 level implies an enlarged lymphatic system, and in diseases associated with lymphoid proliferation, such as lupus nephritis, it is worthwhile to further explore the correlation between sLYVE1 and the disease, which was limited in this study by the small number of lupus patients.

In addition to being an independent predictive marker for MSF and SF, we showed here that sLYVE1 also contributes to the diagnostic performance of traditional clinical markers. Gender, NS-proteinuria, and hypoalbuminemia were also included in the model. The model 2 we developed highlighted sLYVE1 as a crucial component that determines MSF and SF. Proteinuria represents a breakdown of the glomerular barrier. Excess protein is absorbed by the tubular epithelium; however, when this mechanism is overwhelmed, a series of inflammatory and subsequent pro-fibrotic responses are abnormally activated. Studies in a large population-based cohort indicated that a 4-fold increase in albuminuria was associated with a 3-fold increase in the subsequent risk of kidney failure, whereas a 4-fold decrease in albuminuria was associated with a 0.34-times lower risk of renal failure [29]. Increasing proteinuria always represents a worse prognosis, regardless of the initial type of disease [30]. As can be seen in our research, males also contribute a certain number of points to the nomogram, which is consistent with results obtained in previous studies that women are more susceptible to developing CKD, but the disease tends to progress more rapidly in men [31]. As individual indicators do not provide a satisfactory picture of the disease, a nomogram combining indicators of glomerular function (proteinuria and hypoproteinemia) and sLYVE1, which reflects interstitial changes, may optimize the clinical diagnosis.

The strength of this study lies in the extension of lymphatic vascular system abnormalities to clinical predictive applications, presenting for the first time a new potential predictor of renal fibrosis, sLYVE1. Also, in previous studies exploring renal immune dysfunction, they have tended to focus on specific immune cells, yet widespread aggregation of multiple immune cells is a feature of fibrotic progression in the later stages of renal disease. Therefore, in this study, we explored the relationship between a common pathway for the transport of various immune cells (i.e., lymphatics) and renal fibrosis. And, as an indicator of interstitial health, the proposal of sLYVE1 may compensate for previous clinical indicators that assessed only the glomerulus. Furthermore, this study not only proposes a clinical diagnostic model, but also incorporates the new indicator studied into the clinical model constructed from traditional indicators, making the clinical significance of this new indicator clearer. However, the results of this research should also be interpreted in the context of several limitations. First, the characteristics of retrospective cross-sectional studies limit the exploration of changes in sLYVE1 during dynamic disease transitions. Second, this is a single-center study with a limited sample size, leaving the validation of the model lacking a more rigorous external validation. Also, patients originating from the same region and ethnicity limit the breadth of applicability of our proposed model. Therefore, larger multicenter prospective studies are advocated to further explore the potential of sLYVE1 as a predictor of renal fibrosis.

## Conclusion

Based on our results, we suggest that serum sLYVE1 can predict renal fibrosis, and in combination with traditional clinical indicators may be able to reflect the degree of fibrosis to some extent, thus dynamically detecting disease progression in a non-invasive manner.

### Supplementary information

ESM 1 Supplement figure 1: Flowchart of the research. (PDF 17 kb)

ESM 2 Supplementary Figure 2: (a) Calibration curve of the nomogram without sLYVE1 in the training cohort. (b) Calibration curve of the nomogram with sLYVE1 in the training cohort. (c) Calibration curve of the nomogram without sLYVE1 in the validation cohort. (d) Calibration curve of the nomogram with sLYVE1 in the validation cohort. (PNG 237 kb)

High resolution image (EPS 2014 kb)

ESM 3 Supplementary Figure 3: (a) Calibration curve of the nomogram without sLYVE1 in the training cohort. (b) Calibration curve of the nomogram with sLYVE1 in the training cohort. (c) Calibration curve of the nomogram without sLYVE1 in the validation cohort. (d) Calibration curve of the nomogram with sLYVE1 in the validation cohort. (PNG 240 kb)

High resolution image (EPS 2009 kb)

## Data Availability

The original contributions presented in the study are included in the article; further inquiries can be directed to the corresponding author.
