# Supplementary figures and images for: Serum soluble LYVE1 is a promising non-invasive biomarker of renal fibrosis: a population-based retrospective cross-sectional study
Source: Immunol Res. 2023 Dec 23;72(3):476–89. doi: 10.1007/s12026-023-09448-3 (PMC11217098; doi:10.1007/s12026-023-09448-3)

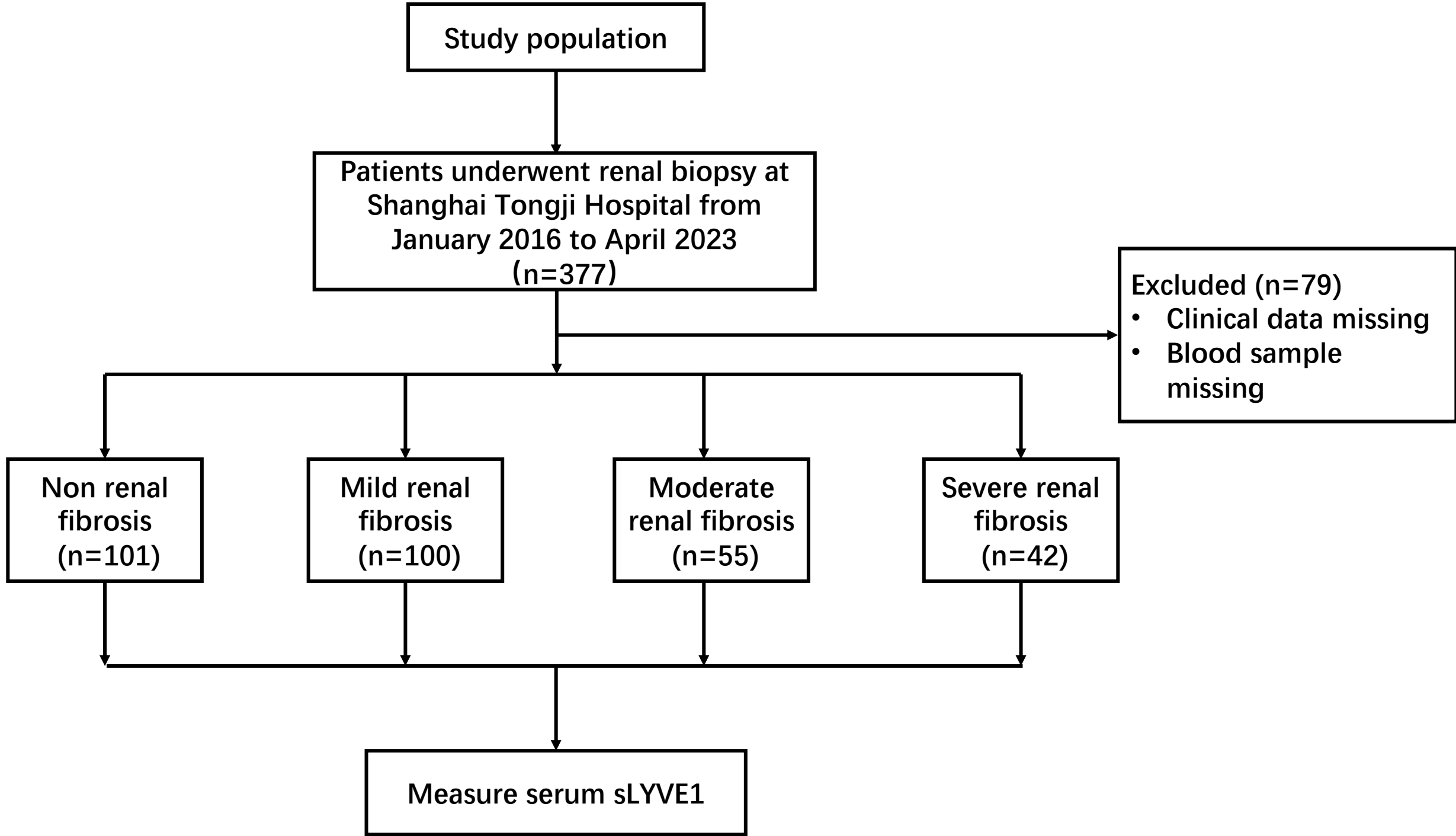

Supplement: Supplementary file 1 — Supplement figure 1: Flowchart of the research. (PDF 17 kb) [file 12026_2023_9448_MOESM1_ESM.pdf]

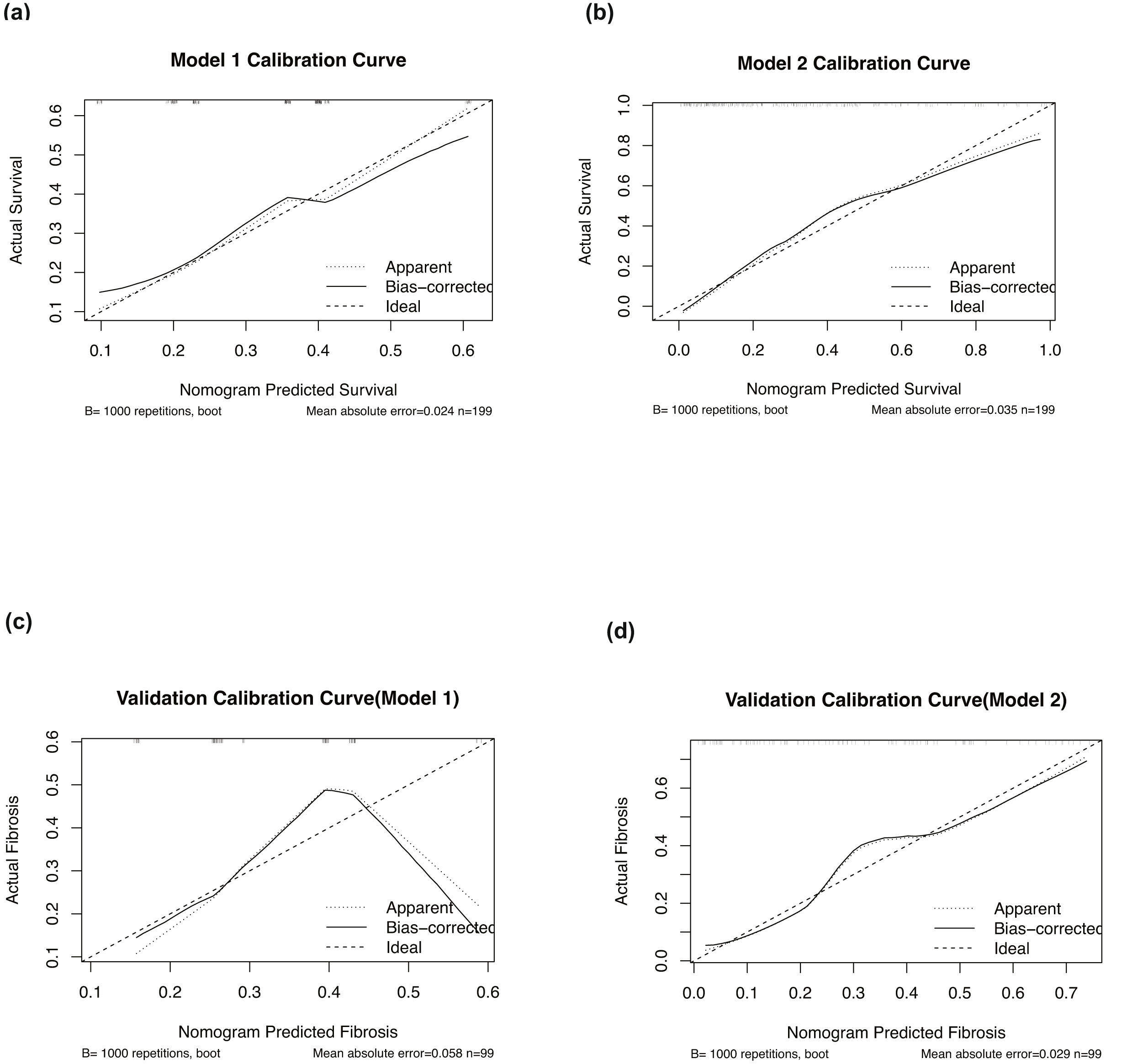

Supplement: Supplementary file 2 — Supplementary Figure 2: (a) Calibration curve of the nomogram without sLYVE1 in the training cohort. (b) Calibration curve of the nomogram with sLYVE1 in the training cohort. (c) Calibration curve of the nomogram without sLYVE1 in the validation cohort. (d) Calibration curve of the nomogram with sLYVE1 in the validation cohort. (PNG 237 kb) [file 12026_2023_9448_Fig6_ESM.png]

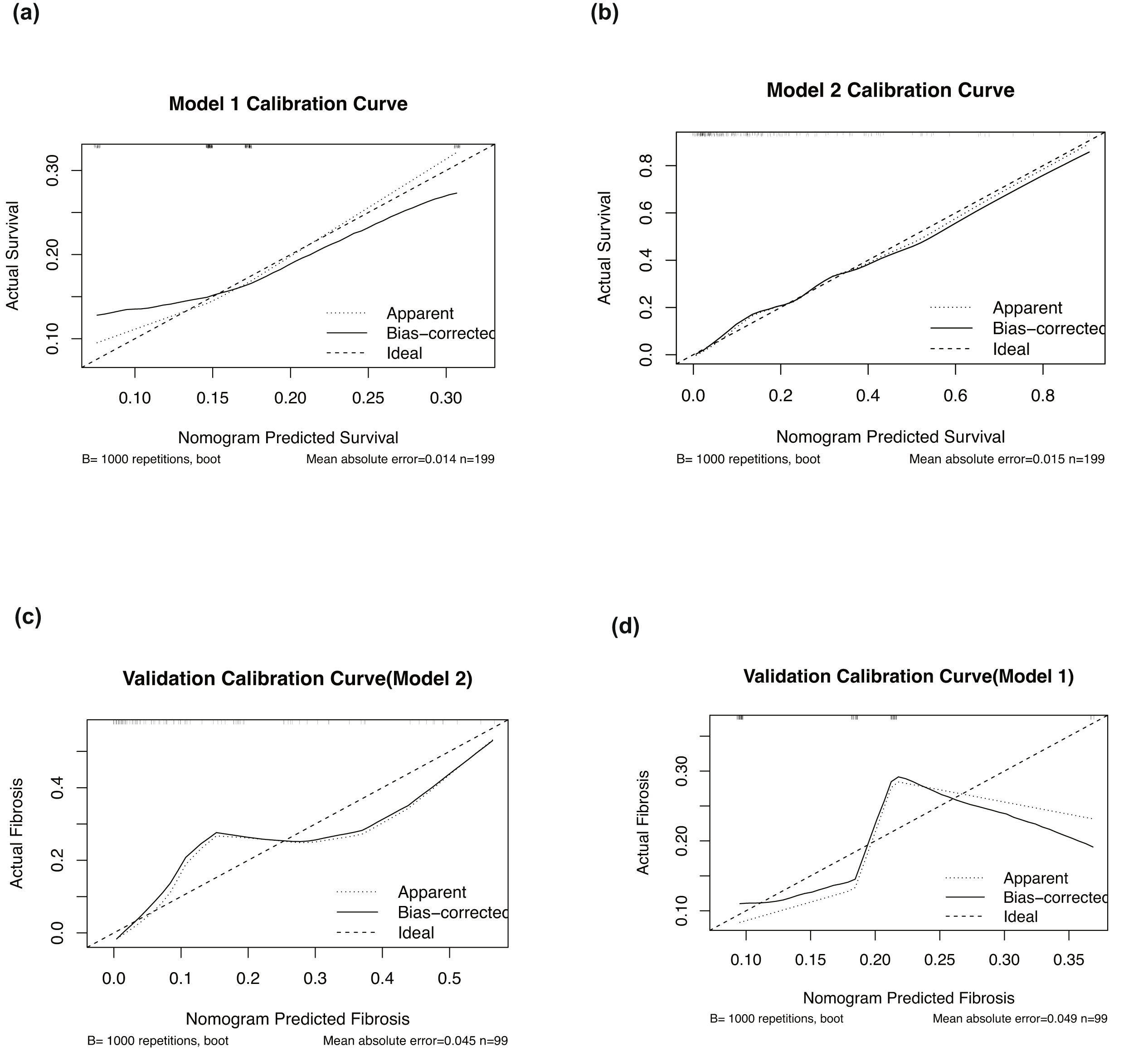

Supplement: Supplementary file 4 — Supplementary Figure 3: (a) Calibration curve of the nomogram without sLYVE1 in the training cohort. (b) Calibration curve of the nomogram with sLYVE1 in the training cohort. (c) Calibration curve of the nomogram without sLYVE1 in the validation cohort. (d) Calibration curve of the nomogram with sLYVE1 in the validation cohort. (PNG 240 kb) [file 12026_2023_9448_Fig7_ESM.png]
